# Ileocecal Intussusception due to a Lipoma in an Adult

**DOI:** 10.1155/2012/684298

**Published:** 2012-09-09

**Authors:** Mehmet Bilgin, Huseyin Toprak, Issam Cheikh Ahmad, Erkan Yardimci, Ercan Kocakoc

**Affiliations:** ^1^Department of Radiology, Medical Faculty, Bezmialem Vakif University, Istanbul, Adnan Menderes Bulvari, Vatan Caddesi, Fatih, 34093 Istanbul, Turkey; ^2^Department of General Surgery, Medical Faculty, Bezmialem Vakif University, 34093 Istanbul, Turkey

## Abstract

While intestinal tumors are rare, small intestinal lipomas are even more uncommon benign neoplasms. They are usually asymptomatic, but lipomas larger than 2 cm may become symptomatic due to obstruction, bleeding, or intussusception. In this paper, US and CT findings of a lipoma located in the terminal ileum and causing ileocecal intussusception were discussed. We report a case of small bowel lipoma that became symptomatic due to intermittent obstruction episodes and ileocecal intussuception. If the diagnosis of intestinal lipoma had been made absolutely as in our case, they should be removed surgically in elective conditions.

## 1. Introduction 

Small intestinal tumors are rare, accounting for 1-2% of all gastrointestinal tract tumors [1]. Among these benign tumors are extremely rare and account for approximately 30% of all small bowel tumors. After gastronitestinal stromal tumors, lipomas consititutes the second most common benign-tumor group [2]. Although they are usually asymptomatic, lipomas larger than 2 cm may cause bowel obstruction, intermittent nonspecific abdominal pain, diarrhea, or bleeding. Furthermore, some lipomas by forming a lead point may cause intussusception, as well [2, 3]. Computed tomography and ultrasonography of the abdomen are helpful radiological modalities for the diagnosis of intestinal lipoma and intussusception caused by this [4, 5]. Intestinal lipomas should be removed because they can cause symptoms such as obstruction or bleeding. Histological evaluation is usually required to exclude the possibility of malignancy in intestinal mass [3].

We described a rare case of symptomatic ileal lipoma associated with ileocecal intussuseption and reviewed some aspects of diagnosis and treatment. 

## 2. Case Presentation

**Figure 1:**
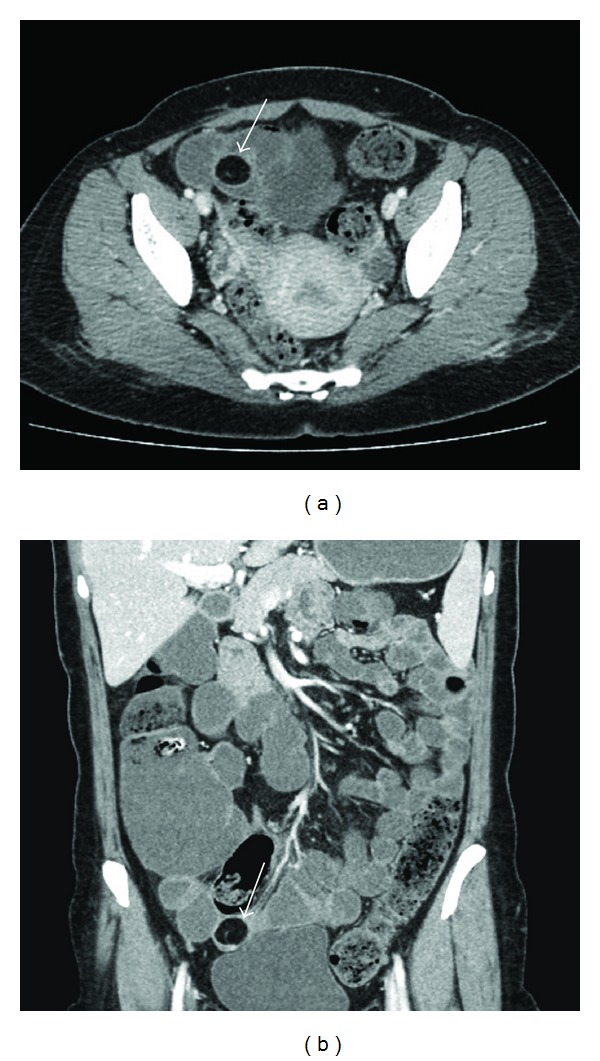
(a) Axial and (b) coronal plan contrast-enhanced CT scans demonstrate a well-circumscribed, intraluminal hypodense 25 × 20 mm mass with fat attenuation (−105 HU) in the terminal ileum (arrow). Any obvious sign of obstruction is not present on this CT.

**Figure 2:**
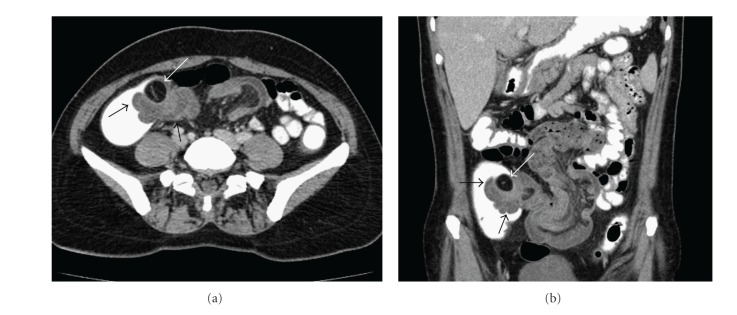
(a) Axial and (b) coronal plan intravenous contrast-enhanced CT scans with oral and rectal contrast demonstrate an ileocolic intussusception with diffuse wall thickening of terminal ileum and the entrance of ileal segment into the cecum at the ileocecal valve level (black arrows). In the cecum adjacent to invaginated segment, hypodense mass lesion consistent with lipoma is seen (white arrow).

**Figure 3:**
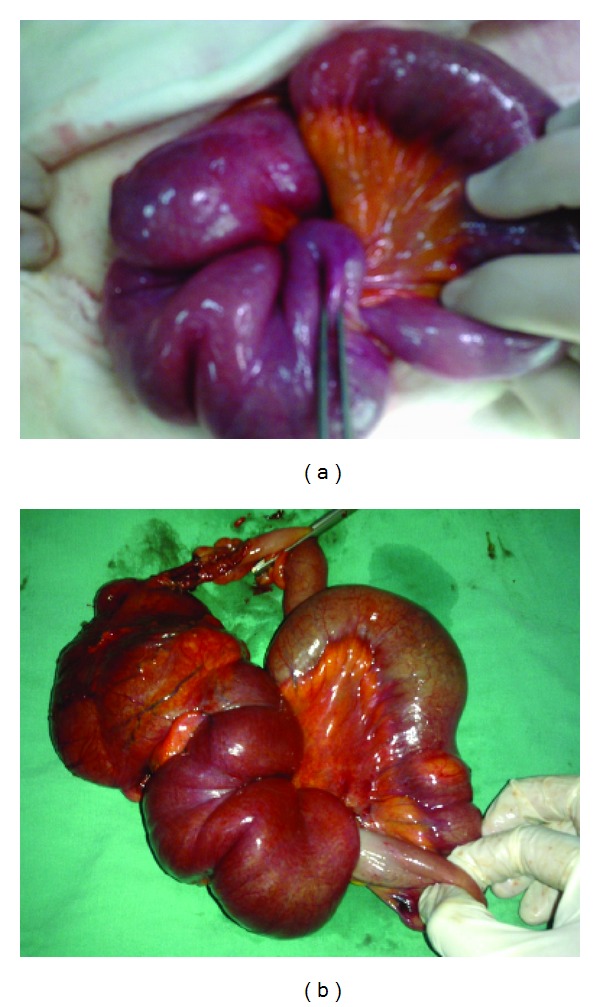
(a) Intraoperative and (b) postsurgical appearence of ileoceacal intususception. Ileocecal resection involving proximal half of the cecum and distal 30 cm of terminal ileum.

39-year-old women was admitted to our emergency clinic with one month history of colicky intermittent abdominal pain and continuous since last 24 hours. Abdominal examination revealed right lower quadrant pain with rebound tenderness. Laboratory findings were unremarkable. In her clinical history, she had been admitted to our hospital again for a day with complaint of nonspecific right lower quadrant pain one month ago. During the stay in our hospital the complaints of the patient had been regressed with nonspecific therapy and she had been sent to home. When early findings, which had been registered in our hospital PACS system, re-examined; a 25 × 20 mm mass close to the ileocecal valve consistent with lipoma was detected on abdominal CT performed during this period (Figures 1(a) and 1(b)). Any obvious sign of obstruction was not present on this CT. Therefore, elective explorative laparotomy had been suggested to the patient, but she had denied an operation. This time, the patient was admitted to our emergency clinic with more severe and prolonged pain. The patient underwent direct abdominal X-ray, abdominal US and CT examination to rule out intestinal pathologies. Abdominal X-ray was unremarkable. Abdominal US examination demonstrated diffuse wall thickening of 15–20 cm segment of terminal ileum, distal to this wall thickening, approximately 5-6 cm “target” appearence. Adjacent to this “target” appearence a 25 × 20 mm well-circumscribed slightly hyperechoic mass lesion was present. Abdominal CT showed diffuse wall thickening of 20 cm of terminal ileum and the entrance of ileal segment into the cecum 5 cm at the ileocecal valve level, representing ileocecal intususception. In the cecum adjacent to intussusceptid segment, a well-circumscribed, homogenously hypodense (−105 Hounsfield Units) (HU) mass lesion consistent with lipoma was seen (Figures 2(a) and 2(b)). Laparoscopic surgery was planned, but during the operation adhesions of small intestine was determined, and the surgeon decided to perform open surgery because of difficulty in exploration. During laparotomic exploration, invagination of approximately 20 cm distal ileum through the ileocecal valve into the cecum and ischemic changes were observed. In the operation, ileocecal resection involving proximal half of the cecum and distal 30 cm terminal ileum and side-to-side ileocolostomy were performed (Figures 3(a) and 3(b)). Histopathological diagnosis was reported as small bowel lipoma located submucosally. The patient was discharged from the hospital on 7th day postoperatively.

## 3. Discussion

Gastrointestinal lipomas are benign tumors that can occur anywhere along the gut in the small bowel. The most common site for lipoma in the small bowel is the ileum [6]. Intestinal lipomas larger than 2 cm may cause complications such as obstruction and bleeding. Intussusception is a common complication of intestinal lipoma [3, 7]. 

Adult intussusception is a rare disease that constitutes approximately 5% of all intussusceptions and accounts for 1% of all adult intestinal obstructions. 3 to 20 per 100.000 of hospital admissions was due to intussusception in adults [4, 6, 7]. Adult intussusception is usually caused by a tumor acting as the apex of the intussusception. Therefore, when the diagnosis of intussusception made, the possibility of the presence of malignancy in the bowel should be kept in mind. However, it has been reported that majority of cases of adult small-bowel intussusception are caused by benign entities, such as lipoma, polyp, Meckel's diverticulum, or adhesions. In both small- and large-bowel intussusception, lipoma is the most common benign tumor [4, 7]. 

Due to the nonspecific and intermittent nature of the symptoms, and difficulty with the examination of the small intestine, preoperative diagnosis is usually difficult [4, 5]. Imaging methods are required to make the diagnosis. Ultrasonography and computed tomography of the abdomen are helpful modalities for the diagnosis of intestinal lipoma and intussusception caused by this. On US, lipoma appears as a round, echogenic mass and pseudokidney sign indicates intussusception [4, 5]. In our case, US demonstrated intussusception with pseudokidney appearence at ileocecal valve level and hyperechoic lipoma in the intussuscepted mass. On CT, lipomas are seen as well-circumscribed, ovoid or round with sharp margins, and homogenous mass. In addition, they demonstrate characteristic attenuation values between −40 and −120 HU typical of the fatty composition [3]. The CT findings of intussusception are a mass-like lesion, including the inner intussusceptum, an eccentric fat density mass that represents the intussuscepted mesenteric fat, and the outer intussuscipiens, and this appears as a “target” or a “sausage” mass according to imaging plane [6]. 

The treatment for small bowel lipomas depends on the clinical manifestations and size. It is not clear whether asymptomatic small lipomas require any intervention, but conservative treatment is often indicated [8]. Surgical resection is indicated if lipomas are symptomatic or to rule out the liposarcomas by performing their histological examination [3]. Elective explorative laparotomy and laparoscopic-assisted resection of lipomas is the best approach because it is less invasive than conventional open surgery. Immediate surgical intervention is mandatory in cases of obstruction, massive hemorrhage, or intussusception [3]. The length of small bowel involved in the intussusception varies from a few centimeters to a meter. If reduction has not been successful, operative resection of a long segment of intussusception requires the excision of a long segment of small bowel. Before resection, intraoperative reduction can be attempted safely to avoid the unnecessary excision of healthy bowel and this method is now a widely accepted method [9]. The type of surgical intervention depends on the patient's medical history and intraoperative findings [3, 10]. 

In conclusion, intussusception is a common complication of intestinal lipoma. We demonstrated a lipoma, located in the terminal ileum, later which caused ileocecal intusussception by US and CT. Intestinal lipomas are benign tumors in which definite diagnosis can be made by their specific appearences on CT. Lipomas should be removed with minimal invasive surgical operation when they are first identified due to possible complications. After the development of complications such as intussusception, resections involving large segments of bowel may be required in emergency conditions.
